# Copper Modulates Mitochondrial Oxidative Phosphorylation to Enhance Dermal Papilla Cells Proliferation in Rex Rabbits

**DOI:** 10.3390/ijms23116209

**Published:** 2022-06-01

**Authors:** Fan Li, Hongli Liu, Xiaojing Wu, Mengqi Liu, Zhengkai Yue, Lei Liu, Fuchang Li

**Affiliations:** 1 College of Animal Science and Technology, Shandong Agricultural University, Tai’an 271018, China; sdaufanli@126.com (F.L.); lhl920216@sdau.edu.cn (H.L.); w963639513@163.com (X.W.); koieliu@126.com (M.L.); 2019110383@sdau.edu.cn (Z.Y.); 2 Hebei Key Laboratory of Specialty Animal Germplasm Resources Exploration and Innovation, Department of Animal Science and Technology, Hebei Normal University of Science and Technology, Qinhuangdao 066004, China

**Keywords:** copper, OXPHOS, UPLC–MS, dermal papilla cell, citric acid

## Abstract

Copper (Cu) is an important coenzyme factor in cell signaling, such as cytochrome c oxidase (Complex IV). Metabolism plays an important role in regulating the fate of mammalian cells. The aim of this study is to experimentally investigate the effect of copper on cell metabolism in the dermal papilla cells of the Rex rabbit. In this study, Cu promoted proliferation of dermal papilla cells (*p* = 0.0008) while also increasing levels of cellular CIII, CIV, Complex IV and ATP. Moreover, fifty metabolites that were significantly different between Cu and controls were identified as potential biomarkers of Cu stimulation. Copper-stimulated cells had altered levels of arachidonic acid derivatives, S-glutamic acid, and citric acid, which were primarily linked to two different pathways: arachidonic acid metabolism (*p* < 0.0001) and alanine, aspartate and glutamate metabolism (*p* = 0.0003). The addition of Cu can increase the proliferation of Rex rabbit dermal papilla cells. Increased levels of ubiquinol-cytochrome c reductase complex core protein 2 (CIII) and cytochrome c oxidase subunit 1 (CIV) were associated with the increased levels of cellular cytochrome c oxidase (Complex IV) and adenosine triphosphate (ATP). In a word, copper promotes cell proliferation by maintaining the function of the cellular mitochondrial electron transport chain (ETC) pathway.

## 1. Introduction

Copper (Cu) has long been used as a growth stimulant; it can stimulate animal appetite and increase feed intake [1] as well as increase the digestibility of crude fat and lipolysis [2]. Dietary Cu supplementation can also improve the fur quality of fox [3] and mink [4]. In general, Cu is essential in all life-forms for the growth and development of multiple types of cells, tissues, and organs. Copper is involved in enzyme reactions as Cu-proteins (e.g., ceruloplasmin), Cu-containing enzymes (e.g., Cu–Zn superoxide dismutase, cytochrome c oxidase), or as a hormone (insulin) [5,6]. However, Cu can also directly regulate the metabolic state of cells through signal molecules [7]. Copper has direct regulatory roles in cyclic AMP-dependent lipolysis in 3T3-L1 adipocytes [8] and the mitogen-activated protein kinase pathway in tumor growth [9]. Moreover, copper is essential for the maintenance of the function of the mitochondrial electron transport chain (ETC); cytochrome c oxidase (Complex IV) is a metalloenzyme at the end of the mitochondrial respiratory chain, and three Cu-containing subunits encoded by mitochondria form the catalytic core of Complex IV [10]. In the absence of Cu ion, Complex IV subunit 1 (CIV) is rapidly degraded followed by failure of the Complex IV assembly; eventually, electron transfer in the respiratory chain is blocked, and synthesis of ATP is reduced [11,12,13]. According to Ramchandani [13], treatment with tetrathiomolybdate, a copper-chelating agent, significantly reduces ATP-linked respiration.

The dermal papillae (DP) are at the bases of hair follicles (HFs). They are composed of specialized mesenchymal cells [14] and are important in the development of mammal HFs. Mammalian dorsal skin HFs undergo a cycle of anagen, catagen, and telogen phases [15]. At the beginning of the anagen stage, signals emanating from DP activate quiescent hair follicle stem cells to produce progenitor cells, which subsequently differentiate into outer root sheath cells to supply the cells needed for hair follicle downgrowth [15]. In addition, the transition of hair follicles from telogen to anagen phases also depends on DP signals. Deficiencies in proteins, trace elements, essential fatty acids, and energy can lead to abnormal hair follicle structure, changes in pigment, or hair loss [14]. In addition to maintaining normal cytochrome c oxidase activity, Cu is also important for aminoxydases, which are essential for keratin fiber strength [16].

Cell metabolism is a complex network involving thousands of biochemical reactions, which is an indispensable link in cell growth, proliferation and responses to extracellular signals. In general, cell proliferation and differentiation are associated with changes in the metabolic program, characterized by a transition from glycolytic metabolism to oxidative phosphorylation (OXPHOS) [15]. According to Ramchandani, [13] Cu depletion leads to a decrease in the level of Complex IV subunits 1 and results in a functional deficiency of the mitochondrial electron transport chain (ETC). In fact, flexible metabolic changes (between glycolysis and OXPHOS) are essential in regulating cyclic changes and development in HFs [15]. Therefore, in this study, Ultra Performance Liquid Chromatography Tandem Mass Spectrometry (UHPLC–MS) was used to investigate metabolic changes in Cu-stimulated dermal papilla cells of the Rex rabbit in order to identify direct links between Cu-mediated metabolic reprogramming and dermal papilla cells proliferation.

## 2. Results

### 2.1. Copper Increases ATP Levels through Cytochrome c Oxidase

**Figure 1:**
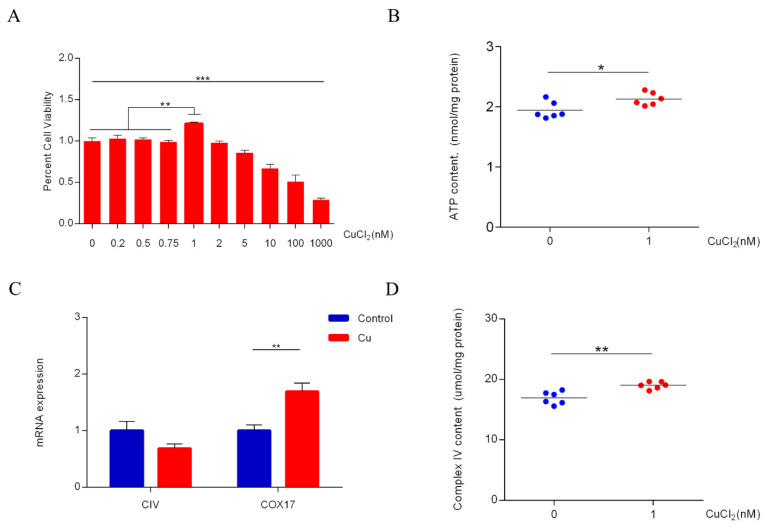
(**A**) CCK-8 assay for cell viability with increasing copper concentration; dermal papilla cells were serum starved in DMEM overnight followed by addition of different amounts of CuCI_2_ for 24 h (*n* = 6). Significance was calculated using One-way ANOVA with Tukey pos*t*-test for comparing multiple groups. * *p* < 0.05, ** *p* < 0.01, *** *p* < 0.001. (**B**) Measurement of intracellular adenosine triphosphate (ATP) content; dermal papilla cells were serum starved in DMEM overnight followed by addition of CuCI_2_ for 24 h (*n* = 6). Analysis was performed by unpaired two-sided *t*-test. * *p* < 0.05. (**C**) qPCR analysis showing transcript levels of cytochrome c oxidase subunit 1 (CIV) and cytochrome c oxidase copper chaperone (COX17) in control and copper (1 nM)-treated dermal papilla cells, treated with copper for 1 h (*n* = 6). Analysis was performed by unpaired two-sided *t*-test. ** *p* < 0.01. (**D**) Measurement of intracellular Complex IV content; dermal papilla cells were serum starved in DMEM overnight followed by addition of CuCI_2_ for 24 h (*n* = 6). Analysis was performed by unpaired two-sided *t*-test. ** *p* < 0.01.

**Figure 2:**
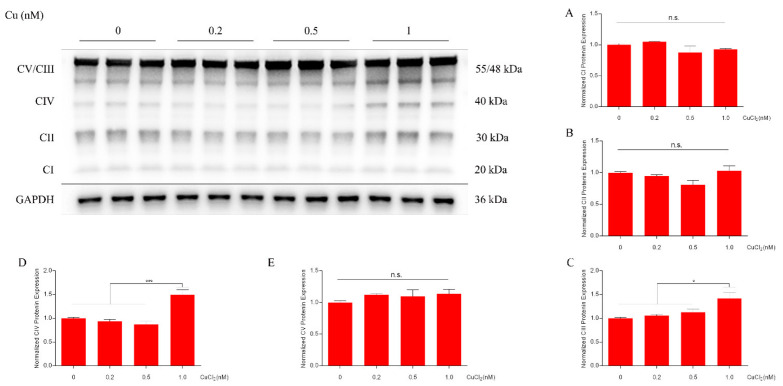
Western blot showing respiratory complexes in dermal papilla cells following 24 h Cu treatment (0, 0.2, 0.5, 1 µM). Complexes I–V are referred to as CI (**A**) (mol. wt. ~19 kDa), CII (**B**) (mol. wt. ~30 kDa), CIII (**C**) (mol. wt. ~48 kDa), (**D**) CIV (mol. wt. ~40 kDa), and (**E**) CV (mol.wt. ~55 kDa), respectively. Significance was calculated using one-way ANOVA with Tukey pos*t*-test for comparing multiple groups. n.s. represents no significant difference. * *p* < 0.05, *** *p* < 0.001.

Cu (1 nM) significantly promoted proliferation of DPCs; with increased Cu concentration (5–1000 nM), Cu significantly inhibited proliferation of DPCs (Figure 1A). Cu (1 nM) also significantly increased intracellular ATP levels (Figure 1B). Because the catalytic core of Complex IV consists of three mitochondria-encoded subunits (containing three Cu atoms), further tests revealed that copper significantly increased cytochrome c oxidase Cu chaperone (COX17) mRNA expression level (Figure 1C), indicating an increase in Cu translocation to mitochondria, but no significant effect on mRNA expression of CIV (Figure 1C). Moreover, copper significantly increased intracellular Complex IV content (Figure 1D). In addition, western blot analysis confirmed an increase in CIII (Figure 2C) and CIV (Figure 2D). Together, these results suggest that Cu-mediated maintenance of mitochondrial function promotes the proliferation of DPc. Therefore, copper doses of 1nM were selected for the subsequent untargeted metabolomics.

### 2.2. Metabolites

**Figure 3:**
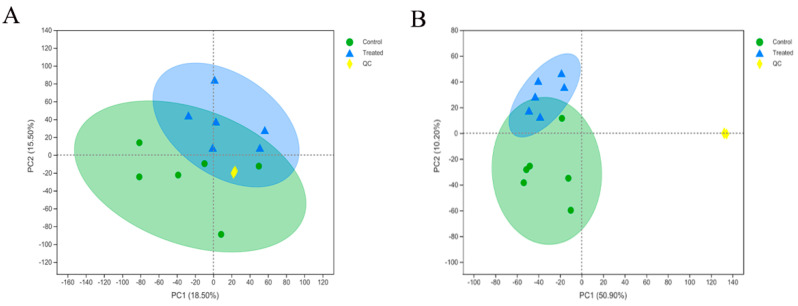
Principal component analysis (PCA) score plot of control; treated and QC samples under positive ion mode (POS) (**A**) and negative ion mode (NEG) (**B**). PCA based on the UHPLC-MS/MS spectra of quality control (QC) samples and metabolites in dermal papilla cells obtained from the control group and treated group (copper group) (*n* = 6).

The purpose of this study was to identify Cu-induced metabolic alterations (control vs treated) in DPCs of Rex rabbits. A total of 19,593 peaks (positive: 9855; negative: 9738) were detected in the LC/MS analysis. A total of 472 metabolites (positive: 231; negative: 241) were identified. In the PCA, QC samples were grouped together, indicating high reproducibility and instrument stability throughout the run (Figure 3A,B).

### 2.3. Multivariate Data Analysis

**Figure 4:**
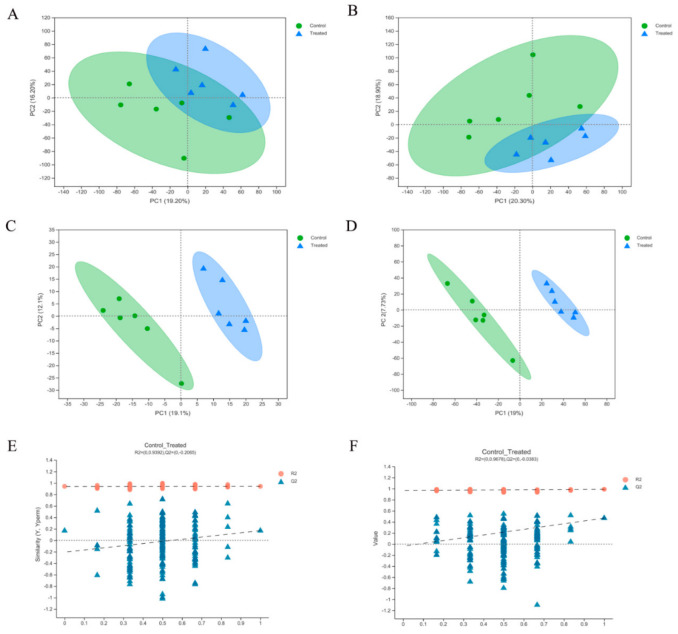
PCA scores of total samples under the positive ion mode (**A**) and negative ion mode (**B**), respectively (*n* = 6). Partial least squares discriminant analysis (PLS-DA) with a 2D score plot of copper group and controls under the positive ion mode (**C**) and negative ion mode (**D**), respectively. Permutation analysis plotting R2 and Q2 from 200 permutation tests in the PLS-DA model, under the positive ion mode (**E**) and negative mode (**F**), respectively. Treated represented copper groups.

Principal component analysis models were constructed in positive ion and negative ion modes, and the first component (PC1) explained 19.2% and 20.3% of the variance, respectively (Figure 4A,B). Differences between groups were determined using PLS-DA, and the large separation between control and treated groups indicated a significant classification effect (Figure 4C,D). According to a previous study [17], the PLS-DA model was tested with response permutation testing. The intercept between the regression line of Q2 and the y-axis was less than 0, which indicated that the model was valid and reliable and there was no excess fitting (Figure 4E,F).

### 2.4. Significantly Different Metabolites

**Figure 5:**
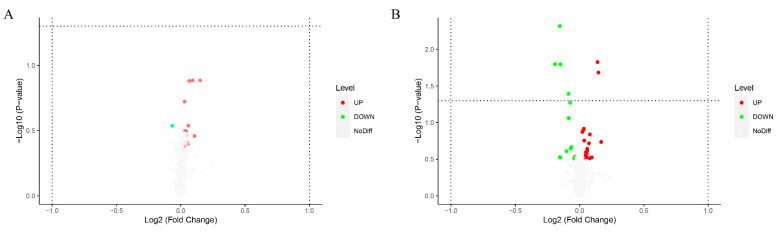
Volcano plot by metabolomics analysis under positive ion mode (**A**) and negative ion mode (**B**), respectively (*n* = 6). The red points represent significantly up-regulated metabolites (*p*-value < 0.05 and VIP > 1) according to the univariate statistical analysis; the green points represent significantly down-regulated metabolites (*p*-value < 0.05 and VIP > 1) and the gray points represent metabolites that did not change.

**Figure 6:**
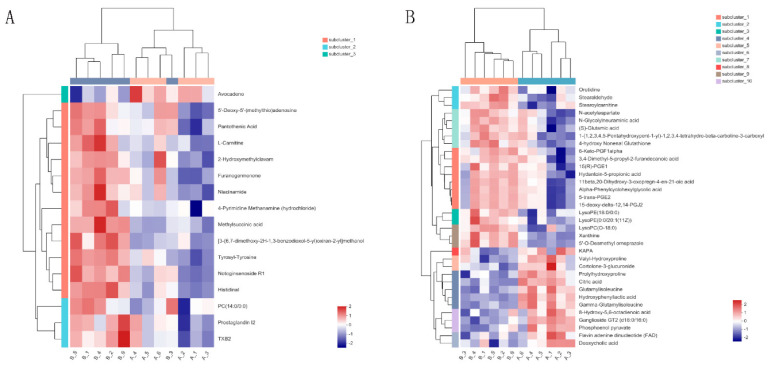
Heat maps of significantly different metabolites under positive ion mode (**A**) and negative ion mode (**B**), respectively (*n* = 6). The color in the panel represents the relative levels of each metabolite: purple represents low levels, and red represents high levels. In maps, class (**A**) represented control samples and class (**B**) represented copper samples.

**Figure 7:**
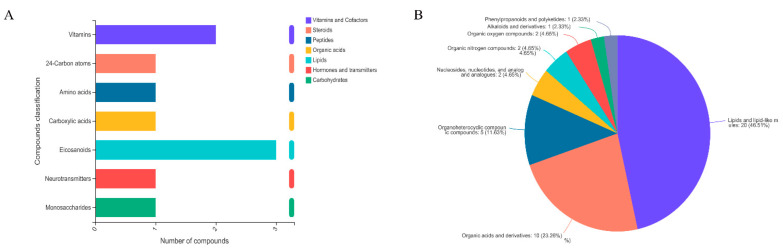
Classification analysis of differential metabolites based on KEGG database (**A**) and HMDB database (**B**).

Significant differences in metabolites between control and Cu-treated groups were determined using PLS-DA and Student’s *t*-tests. According to a previous study, [18] the criteria were *p* < 0.05 and VIP > 1. Fifty significantly different metabolites were identified (Table 1 and Table 2). Figure 5 shows volcano plots, with one point representing a metabolite. There were 15 and 21 metabolites significantly up-regulated and 1 and 13 metabolites significantly down-regulated in positive ion (POS) (Figure 5A) and negative ion (NEG) modes (Figure 5B), respectively. Hierarchical clustering analysis was conducted to explore expression patterns of differential metabolites (Figure 6). Last, differential metabolites were classified using the KEGG and HMDB databases (Figure 7). Ten metabolites were matched and classified into the primary seven categories, with three of those metabolites included in eicosanoids (Figure 7A). Forty-three metabolites were matched and classified into eight HMDB superclasses, with 20 of those metabolites included in the “lipids and lipid-like molecules” term (Figure 7B).

### 2.5. Kyoto Encyclopedia of Genes and Genomes’ Functional Pathways

**Figure 8:**
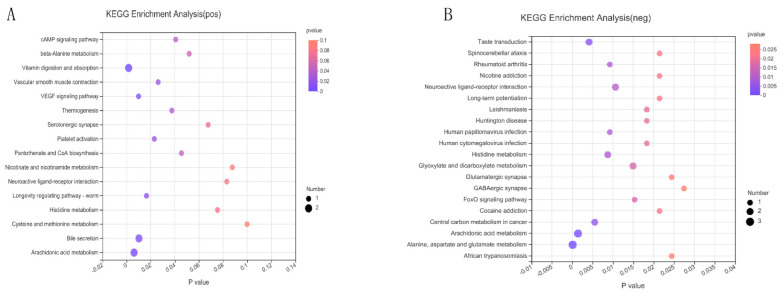
Differential metabolomic pathways of differentially expressed metabolites according to the KEGG database matched results under positive ion mode (**A**) and negative ion mode (**B**), respectively (*n* = 6). The size of each point represents the metabolite number, and the color represents *p*-value.

To explore the potential pathways of differential metabolites, metabolic pathway enrichment analysis was performed based on the KEGG database under positive and negative ion modes (Figure 8). Copper stimulation significantly affected arachidonic acid metabolism and alanine, aspartate and glutamate metabolism.

## 3. Discussion

Copper is a redox-active transition metal and is generally regarded as a static enzyme cofactor [8]. Recent studies have revealed the usefulness of Cu in the development of mammalian HFs. Zhang [19] reported that Cu effectively induces proliferation and differentiation of skin and HF-related cells, and 19 mg Cu/kg of dry matter (DM) increases the number of active secondary follicles [20]. Cell proliferation is generally associated with increases in metabolic requirements, [15] including for amino acids, fatty acids, and especially energy (ATP). In this study, the suitable concentration of Cu for DPCs was determined using Cell-Counting-Kit-8 (CCK-8). Copper at 1 nM promoted proliferation of DPCs (Figure 1A). Moreover, starting from a concentration of Cu of 5 nM, Cu significantly inhibited proliferation of DPCs; in fact, it is not uncommon with Cu and other metals to see increased proliferation at low concentrations followed by a sudden drop when the dose becomes cytotoxic. Cu stimulation also increased intracellular levels of ATP (Figure 1B). Here, qRT-PCR analysis confirmed an increase in COX17 mRNA expression (Figure 1C), indicating an increase in copper transport from the cytoplasm to the mitochondria. As shown in Figure 1D, copper increased the protein of Complex IV without impacting the mRNA level of CIV (Figure 1C), which is consistent with the study of Ramchandani [13]. Moreover, western blot analysis showed an increase in CIV (Figure 2D), a subunit of Complex IV. Horn [10] reported that three mitochondria-encoded subunits (CIV, MTCO2, and MTCO3) formed the catalytic core of Complex IV, containing three copper atoms; MTCO2 combines with two Cu atoms to form the Cu_A_ site and the heme *a*_3_ group of CIV combines with one Cu atoms to form the Cu_B_ site. Therefore, we suggest that copper-mediated CIV subunit expression promotes the assembly of Complex IV and maintains the function of ETC, which is consistent with the result that copper treatment increases the intracellular content of ATP. In fact, generation of energy by OXPHOS is highly efficient compared with that by anaerobic glycolysis [15]. 

To further explore the metabolic changes linked to Cu stimulation in DPCs, we performed untargeted metabolomics using LC-MS. The compounds’ classification was performed on the differential metabolites’ base in the HMDB database and KEGG database. Forty-three differential metabolites were classified in eight categories (Figure 7B). Of those metabolites, 20 metabolites were classified as lipids and lipid-like molecules, and 10 were classified as organic acids. Copper has long been associated with lipid metabolism. It has a positive role in lipid metabolism [2,9] can increase lipolysis in the liver and adipose tissue, and can promote fatty acid uptake in skeletal muscle. The results in this study also indicated high correlation between Cu and lipid metabolism in DPCs. 

**Figure 9:**
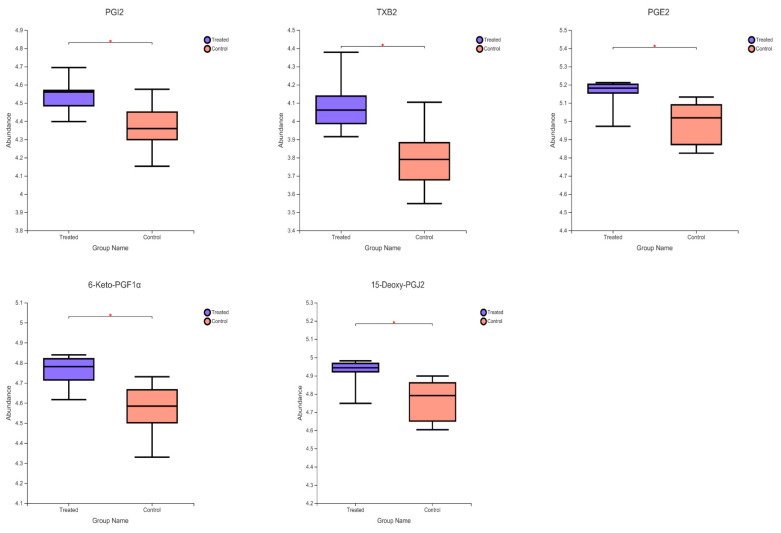
Relative abundance of arachidonic acid derivative prostaglandin I2 (PGI2), thromboxane (TXB2), 5-trans-PGE2 (PGE2), 6-Keto-prostaglandin F1alpha (6-Keto-PGF1α) and 15-deoxy- delta-12,14-PGJ2 (15-Deoxy-PGJ2) (*n* = 6). * *p* < 0.05. In maps, control represented control group and treated represented copper group.

Pathway enrichment analysis was performed on differential metabolites to determine the most relevant pathways associated with Cu stimulation. Arachidonic acid metabolism (*p* < 0.0001), Histidine metabolism (*p* = 0.0013) and alanine, aspartate and glutamate metabolism (*p* = 0.0003) were the most significantly enriched pathways (Figure 8). In this study, the contents of prostaglandin I2 (PGI2), thromboxane (TXB2), 5-trans-PGE2 (PGE2), 6-keto-prostaglandin F1 alpha (6-keto-PGF1α), and 15-deoxy-delta-12,14-PGJ2 (15-Deoxy-PGJ2) increased significantly (Figure 9). Arachidonic acid is an ω-6 polyunsaturated fatty acid that is commonly found in mammalian cells, and has important biochemical roles, primarily as the direct precursor of many biologically active eicosanoic acid derivatives [21]. An increase in arachidonic acid and the concomitant production of eicosanoids can promote cell proliferation [22]. Arachidonic acid is released via cell membrane phospholipids catalyzed by phospholipase A2 in response to various physiological and pathological stimuli. Under the action of cyclooxygenase, it is converted to prostaglandin intermediate metabolites PGG2 and PGH2, in turn [23]. Various biologically active prostaglandins such as PGI2, PGE2, PGF2α, PGD2, and thromboxane A2 (TXA2) are produced by the action of different downstream prostaglandin synthases [24]. The most important metabolite is PGE2, because it has an irreplaceable role in cell proliferation [25]. The physiological function of PGE2 is achieved by activating its homologous G protein-coupled E prostaglandin receptor (EP1–EP4) [26]. According to Liu, [27] PGE2 can specifically activate the classical Wnt/β-catenin signaling pathway. In this study, the Cu-induced proliferation of DPCs (Figure 1A) might have been associated with increased levels of intracellular PGE2. In addition to PGE2, PGI2 is also one of the most important metabolites. Similar to TXA2, PGI2 contains epoxide bonds that can rapidly hydrolyze and have a short half-life of 30 s [22]. In this study, the contents of PGJ2 and 6-keto-PGF1α (final metabolite of PGJ2) also increased significantly. The compound PGI2 stimulates cAMP production via adenylate cyclase (AC) [8]. Krishnamoorthy [8] reported that Cu increases cAMP levels by inhibiting activity of the cAMP-degrading phosphodiesterase PDE3B. However, even if cAMP was regulated by both promotion of cAMP production (PGI2-AC-cAMP) and inhibition of cAMP degradation (PDE3B-cAMP), the absence of changes in cAMP content under Cu stimulation was unexpected. The absence of changes in the level of cAMP might have been due to differences in cell types.

Amino acids are the most important components of proteins and also serve as backup energy sources for cells [28]. Amino acids may also act as signaling molecules to regulate a variety of cellular physiological processes, including proliferation and autophagy [29]. Moreover, the chelation by amino acids is often important for copper homeostasis and storage. Copper homeostasis is maintained by a conserved group of proteins that often contain copper binding domains rich in cysteine, methionine or Histidine residues. [30,31] In monogastric animals, a set of sound regulatory mechanisms maintains Cu homeostasis [1]. Cu^2+^ is normally absorbed by the intestine, then enters blood circulation, and binds to serum albumin and L-histidine that form an exchangeable pool of copper [30]. Due to the high cytotoxicity of copper ions, intracellular copper ions are usually bound to specific copper chaperone proteins (e.g., cytochrome c oxidase 17, anti-oxidant 1, and copper chaperones for superoxide dismutase) and transported to specific target proteins; this is also considered to be a form of storage of copper ions [32]. In this study, Cu stimulation increased the contents of N-acetyl-aspartic acid, and Histidine metabolites (l-Histidinal, Hydantoin-5-propionic acid and S-glutamic acid) (Figure 5). We believe that the increased amino acid production may be due to the chelation by amino acids for copper homeostasis. Glutamic acid has an important role in hair growth and the proliferation of HF cells as an essential nutrient for DP and hair matrix cells. [33,34,35] *N*-acetyl aspartate is an important source of amino acids and acetates in the mammalian brain [36], and it is synthesized from aspartate and acetyl coenzyme A (acetyl-CoA) by the enzyme l-aspartate-*N*-acetyltransferase [37]. Acetyl-CoA is an important intermediate metabolite that is the hub of sugar, lipid, and protein metabolism [38]. The main sources of acetyl-CoA are the oxidative decarboxylation of pyruvate (a product of glycolysis) and the β-oxidation of fatty acids. However, no change in pyruvate and Acetyl-CoA contents were detected in this study, although phosphoenolpyruvate content simultaneously decreased significantly (Figure 5). The expression of CIV subunit (Figure 2D) due to Cu addition significantly increases the content of cytochrome c oxidase (Figure 1C), which ultimately leads to the attenuation of glycolysis accompanied by increased OXPHOS [8]. In this study, cytochrome c oxidase content increased significantly (Figure 1C), which is consistent with the study of Ramchandani [8]. However, Cu did not affect the mRNA expression of CIV (Figure 1D). In addition, Cu increased the mRNA expression of COX17, indicating an increase in Cu translocation from mitochondria to cytochrome c oxidase (Figure 1D). Thus, the decrease in phosphoenolpyruvate content was attributed to increased OXPHOS and weakened glycolysis. Furthermore, it is worth nothing that citric acid content decreased (Figure 5). The citric acid cycle (TCA cycle) is the final common oxidative pathway for carbohydrates, fats, and amino acids [39]. The first step in the TCA cycle is the condensation of acetyl-CoA and oxaloacetate to citric acid by the action of citrate synthase (CS) [40]. Metal ions such as Cu^2+^, Zn^2+^, and Mg^+^ have strong inhibitory effects on CS activity [41]. In addition, ATP is also an allosteric inhibitor of CS [42]. Therefore, inhibition of CS by Cu^2+^ and increased levels of ATP might explain the significant decrease in citric acid content.

## 4. Materials and Methods

### 4.1. Cells

Dermal papilla cells (DPCs) of Rex rabbits were kindly provided by Professor Xinsheng Wu (College of Animal Science and Technology, Yangzhou University, Jiangsu, China) and were identified as described previously [43].

### 4.2. Cell Culture

Dermal papilla cells were cultured at 37 °C in a cell incubator with 5% CO2. For the CCK-8 experiment, DPCs were serum starved in Dulbecco’s modified eagle medium (DMEM) (Thermo Fisher, Carlsbad, CA, USA) with 2% Fetal Bovine Serum (FBS) (Thermo Fisher, Carlsbad, CA, USA) overnight. Cells were then treated with 0, 0.2, 0.5, 0.75, 1.0, 2, 5, 10, 100, 1000 nM Cu (as copper chloride) for 24 h in DMEM with no serum. After treatment, cells were washed with PBS twice and then 200 µL of lysate was added to each well of a 6-well plate, and then the lysate was collected into a 1.5 mL centrifuge tube followed by centrifugation at 12,000× *g* for 5 min. Then, contents of ATP and Complex IV were determined using a Varioskan LUX plate reader (Thermo Fisher, Carlsbad, CA, USA). Solarbio provided a Cell Proliferation and Cytotoxicity Assay Kit (CCK-8) (CA1210; Beijing Solarbio Science & Technology Co., Ltd., Beijing, China); Beyotime supplied an ATP Assay Kit (S0026; Shanghai Beyotime Science & Technology Co., Ltd., Shanghai, China); and Enzyme-linked Biotechnology supplied a Complex IV assay kit (Shanghai Enzyme-Linked Biotechnology Co., Ltd., Shanghai, China). Simultaneously, the protein content of each sample was measured with a protein content assay kit (Thermo Fisher, Carlsbad, CA, USA). Contents of ATP and Complex IV were normalized to nmol/mg protein.

### 4.3. RNA Isolation and Analysis

Extraction of total RNA and reverse-transcription quantitative PCR were performed as described previously. Accurate Biotechnology supplied a reverse-transcription kit (Accurate Biotechnology Co., Ltd., Hunan, China) and Takara supplied SYBR Green master mix (Takara, Dalian, China). The gene for normalization was GAPDH (glyceraldehyde-3-phosphate dehydrogenase), and results of relative mRNA quantification were verified using β-actin levels. The mRNA expression was analyzed using the 2^-ΔΔCT^ method. [1,2] Primer sequences are shown in Table 3.

### 4.4. Western Blot

Total protein was extracted using a radioimmunoprecipitation assay (RIPA) lysis buffer (Solarbio, Beijing, China) with addition of PMSF and protease inhibitor; the protein concentration was determined using a BCA assay kit (Beyotime, Shanghai, China). The proteins were separated on a 7.5–10% SDS polyacrylamide gel electrophoresis, transferred on PVDF membranes at 200 mA at 4 °C and closed in fast closing solution (New Cell Molecular Biotechnology Co., Ltd., Shanghai, China) for 30 min. Protein detection was performed using enhanced chemiluminescence detection reagents (Beyotime, Shanghai, China). Polyclonal rabbit anti-GAPDH antibody was used as a loading control. Western blots were developed and quantified with BioSpectrum 810 Imaging System using VisionWorksLS 7.1 software (UVP LLC, Upland, CA, USA). The standard markers for protein molecular masses were supplied by Thermo (Cat# 26617, Thermo Fisher, Carlsbad, CA, USA). The membranes were probed with the required antibodies: OXPHOS antibody cocktail (Cat# ab110413, abcam, Cambridge, UK), GAPDH (Cat# ab9485, abcam, Cambridge, UK). The horseradish peroxidase (HRP)-conjugated goat anti-rabbit IgG and goat anti-mouse IgG antibody were supplied by Beyotime (Beyotime, Shanghai, China).

### 4.5. Metabolite Extraction

Cell samples were added to 2-mL centrifuge tubes. Grinding beads (6 mm) and 400 µL of extract solution (methyl alcohol:water = 4:1) containing 0.02 mg/mL L-2-chlorophenylalanine were added to the tubes. Tubes were transferred to a frozen tissue grinder for 6 min (−10 °C, 50 Hz). Metabolites were extracted for 30 min (5 °C, 40 KHz) and then left for 30 min (−20 °C). Last, samples were centrifuged for 15 min (13,000× *g*, 4 °C). In addition, 20 µL of supernatant from each sample was mixed well and used as a quality control sample.

### 4.6. UHPLC–MS Analysis

The UHPLC–MS/MS testing was conducted by Shanghai Majorbio Bio-pharm Technology Co., Ltd. (Majorbio, Shanghai, China). The LC–MS/MS analyses were performed using a Vanquish UHPLC system (Thermo Fisher, Carlsbad, CA, USA) coupled with an Orbitrap Q Exactive series mass spectrometer (Thermo Fisher, Carlsbad, CA, USA). Samples were injected onto an ACQUITY UPLC HSS T3 column (100 mm × 2.1 mm i.d., 1.8 μm) using a 16-min linear gradient at a flow rate of 0.2 mL/min. Mobile phase A was 95% water + 5% acetonitrile (including 0.1% formic acid), and mobile phase B was 47.5% acetonitrile + 47.5% isopropanol + 5% water (containing 0.1% formic acid). The flow rate was 0.40 mL/min, the injection volume was 2 μL, and the column temperature was 40 °C. The Q Exactive series mass spectrometer was operated in positive and negative polarity modes with spray voltage of 3.5 kV and −2.9 kV, respectively, capillary temperature of 320 °C, sheath gas flow rate of 40 arb, aux gas flow rate of 10 arb, and resolution of 17,500 MS2.

### 4.7. Database Search

Raw data files generated by LC/MS were processed using Progenesis QI (Waters, Milford, CT, USA) to perform peak alignment, peak selection, and quantitation of each metabolite. Peaks with a relative standard deviation greater than 30% in QC samples were excluded. Last, peak intensities were normalized to the total spectral intensity.

### 4.8. Data Analysis

Metabolites were annotated using the Kyoto Encyclopedia of Genes and Genomes (KEGG) database (http://www.genome.jp/kegg/), Human Metabolome Database (HMDB) (www.hmdb.ca/), and Lipidmaps database (http://www.lipidmaps.org/). Principal component analysis (PCA) and partial least squares discriminant analysis (PLS-DA) were performed using the online Majorbio Cloud Platform (www.majorbio.com). Statistical significance (*p*-value) was determined using univariate analysis (*t*-test). Metabolites with VIP_PLS-DA > 1 and *p* < 0.05 were considered to be differential metabolites. Volcano plots were prepared at BIC (http://www.ehbio.com/Cloud_Platform/front/#/), with Log2 (fold change) on the x-axis and −Log10 (*p*-value) on the y-axis. Cluster and functional analyses of metabolites were performed using the online Majorbio Cloud Platform (www.majorbio.com). Metabolic pathways at *p* < 0.05 were considered to be significantly enriched.

## 5. Conclusions

Cu is essential for maintaining the function of the cellular mitochondrial electron transport chain (ETC) pathway and an appropriate concentration of Cu (1 nM) can promote the proliferation of DPc. In addition, we use untargeted metabolomics to provide a direct link between Cu-mediated metabolic reprograming and the proliferation of DPc. In brief, a direct regulatory role of Cu in the cellular metabolism of DPCs was revealed in this study, which will provide an important reference basis for future studies on the biological roles of Cu and highlight its potential to be developed as a metabolic regulatory factor.

## Figures and Tables

**Table 1:** Positively identified significantly differentially expressed metabolites in the dermal papilla cells after copper treatment under positive ion mode (*n* = 6).

Metabolite	Formula	M/Z	RT (Min)	FC	*p*-Value	VIP	Regulate
PC (14:0/0:0)	C22H46NO7P	468.31	7.42	1.02	0.0128	1.21	up
Prostaglandin I2	C20H32O5	353.23	4.75	1.04	0.0394	1.44	up
Niacinamide	C6H6N2O	123.06	1.32	1.02	0.044	1.18	up
5’-Deoxy-5’-(methylthio)adenosine	C11H15N5O3S	298.1	1.85	1.04	0.0093	1.75	up
Pantothenic Acid	C9H17NO5	220.12	1.76	1.03	0.0197	1.38	up
[3-(6,7-dimethoxy-2H-1,3-benzodioxol-5-yl)oxiran-2-yl]methanol	C12H14O6	237.08	3.24	1.02	0.0023	1.06	up
Methylsuccinic acid	C5H8O4	133.05	1.65	1.11	0.0004	2.37	up
4-Pyrimidine Methanamine (hydrochloride)	C5H7N3	110.07	0.64	1.02	0.0479	0.94	up
Histidinal	C6H9N3O	122.07	0.68	1.05	0.0006	1.74	up
L-Carnitine	C7H15NO3	162.11	0.68	1.02	0.0361	1.15	up
Tyrosyl-Tyrosine	C18H20N2O5	309.13	3.48	1.03	0.0139	1.28	up
Furanogermenone	C15H20O2	215.14	6.06	1.04	0.032	1.35	up
Avocadene	C17H34O3	304.28	6.3	0.96	0.0094	1.55	down
TXB2	C20H34O6	393.22	4.75	1.08	0.0186	1.98	up
Notoginsenoside R1	C47H80O18	478.27	2.38	1.07	0.0004	2.07	up
2-Hydroxymethylclavam	C6H9NO3	144.07	0.76	1.02	0.0322	1.06	up

M/Z: mass charge ratio; RT: Retention time; FC: Fold change; VIP: Variable Importance for Projection, one indicator reflecting the capability of the variables to explain Y.

**Table 2:** Positively identified significantly differentially expressed metabolites in the dermal papilla cells after copper treatment under negative ion mode (*n* = 6).

Metabolite	Formula	M/Z	RT (Min)	FC	*p*-Value	VIP	Regulate
5-trans-PGE2	C20H32O5	333.2072	5.4926	1.032478	0.03404	1.23838	up
11beta,20-Dihydroxy-3-oxopregn-4-en-21-oic acid	C22H30O5	419.2053	5.4926	1.039048	0.02904	1.25489	up
Orotidine	C10H12N2O8	333.0592	0.65	1.034972	0.02517	1.268658	up
(S)-Glutamic acid	C5H9NO4	146.0447	0.6788	1.035462	0.03697	1.329292	up
Flavin adenine dinucleotide (FAD)	C27H33N9O15P2	784.1511	1.9715	0.975401	0.0371	1.047502	down
Hydroxyphenyllactic acid	C9H10O4	181.0498	2.1269	0.898861	1.51E-05	2.355815	down
KAPA	C9H17NO3	186.1127	2.9861	0.971601	0.04326	1.199216	down
1-(1,2,3,4,5-Pentahydroxypent-1-yl)-1,2,3,4-tetrahydro-beta-carboline-3-carboxylate	C17H22N2O7	365.1361	1.7469	1.043125	0.02177	1.30918	up
4-hydroxy Nonenal Glutathione	C19H33N3O8S	462.192	3.3535	1.057359	0.005853	1.52711	up
3,4-Dimethyl-5-propyl-2-furandecanoic acid	C19H32O3	307.2279	7.5602	1.057021	0.04261	1.368364	up
LysoPC(O-18:0)	C26H56NO6P	554.383	9.0584	1.02186	0.004398	1.2194	up
Citric acid	C6H8O7	191.0189	1.5058	0.876169	0.000109	2.687449	down
N-Glycolylneuraminic acid	C11H19NO10	324.0935	0.7009	1.05218	0.01219	1.55297	up
Hydantoin-5-propionic acid	C6H8N2O4	343.0911	0.7431	1.031465	0.0479	1.059106	up
Xanthine	C5H4N4O2	151.0251	1.6527	1.107071	0.000208	2.243746	up
5′-O-Desmethyl omeprazole	C16H17N3O3S	368.0461	1.6527	1.101595	9.80E-05	2.193221	up
Prolylhydroxyproline	C10H16N2O4	227.1033	2.0353	0.943273	0.002148	1.599332	down
Cortolone-3-glucuronide	C27H42O11	587.269	2.3248	0.953163	0.01874	1.39429	down
Gamma-Glutamylisoleucine	C11H20N2O5	241.1191	2.6473	0.902102	0.000121	2.100603	down
6-Keto-PGF1alpha	C20H34O6	369.2282	4.7524	1.04205	0.01969	1.421972	up
Deoxycholic acid	C24H40O4	391.2858	7.4298	0.899243	0.03853	1.777803	down
LysoPE(18:0/0:0)	C23H48NO7P	480.3097	8.8337	1.014879	0.005051	0.995126	up
LysoPE(0:0/20:1(11Z))	C25H50NO7P	506.3251	8.8789	1.025503	0.009524	1.055846	up
Stearoylcarnitine	C25H49NO4	464.3147	9.0584	1.023518	0.003941	1.197591	up
Stearaldehyde	C18H36O	313.2748	9.0136	1.123257	0.01091	2.161813	up
15(R)-PGE1	C20H34O5	353.2333	5.5508	1.033359	0.04541	1.076758	up
15-deoxy-delta-12,14-PGJ2	C20H28O3	315.1964	5.4926	1.032549	0.03407	1.213301	up
Alpha-Phenylcyclohexylglycolic acid	C14H18O3	233.1178	5.4926	1.039793	0.04039	1.189628	up
Glutamylisoleucine	C11H20N2O5	241.1191	2.8273	0.942313	0.000573	1.700102	down
Ganglioside GT2 (d18:0/16:0)	C88H154N4O42	968.4949	2.5782	0.95	0.00089	1.774021	down
8-Hydroxy-5,6-octadienoic acid	C8H12O3	467.2263	2.1958	0.901608	0.0406	1.763572	down
Valyl-Hydroxyproline	C10H18N2O4	251.1036	1.7057	0.957097	0.01674	1.273209	down
N-acetylaspartate	C6H9NO5	174.0398	1.2671	1.068341	0.03957	1.420163	up
Phosphoenol pyruvate	C3H5O6P	166.974	0.7656	0.93201	0.02386	1.694889	down

M/Z: mass charge ratio; RT: Retention time; FC: Fold change; VIP: Variable Importance for Projection, one indicator reflecting the capability of the variables to explain Y.

**Table 3:** Primer sequences of related genes.

Genes ^1^	GenBank Accession Number	Primer Sequences (5′–3′)	Product Size/bp
CIV	XM_008252337.2	F: 5′-CTATTTGGAGCTTGAGCTGGGATGG-3′ R:5′-AAGGCATGTGCGGTGACGATTAC-3′	128
COX17	XM_002716664.3	F: 5′-CAGGAGAAGAAGCCGCTGAAGC-3′ R:5′-GGGCCTCAATTAGATGTCCACAGTG-3′	141
GAPDH	NM_001082253.1	F: 5′-CACCAGGGCTGCTTTTAACTCT-3′ R:5′-CTTCCCGTTCTCAGCCTTGACC-3′	163
β-actin	XM_002722894.3	F: 5′-CGCAGAAACGAGACGAGATT-3′ R:5′-GCAGAACTTTGGGGACTTTG-3′	123

^1^ CIV = cytochrome c oxidase subunit 1; COX17 = cytochrome c oxidase copper chaperone; GAPDH = glyceraldehyde phosphate dehydrogenase; β-actin = actin alpha.

## Data Availability

The datasets generated during and/or analyzed during the current study are available from the corresponding author on reasonable request.
